# Levetiracetam Reverses Synaptic Deficits Produced by Overexpression of SV2A

**DOI:** 10.1371/journal.pone.0029560

**Published:** 2011-12-29

**Authors:** Amy Nowack, Erik B. Malarkey, Jia Yao, Adam Bleckert, Jessica Hill, Sandra M. Bajjalieh

**Affiliations:** 1 Department of Pharmacology, University of Washington, Seattle, Washington, United States of America; 2 Graduate Program in Neurobiology and Behavior, University of Washington, Seattle, Washington, United States of America; University of Leicester, United Kingdom

## Abstract

Levetiracetam is an FDA-approved drug used to treat epilepsy and other disorders of the nervous system. Although it is known that levetiracetam binds the synaptic vesicle protein SV2A, how drug binding affects synaptic functioning remains unknown. Here we report that levetiracetam reverses the effects of excess SV2A in autaptic hippocampal neurons. Expression of an SV2A-EGFP fusion protein produced a ∼1.5-fold increase in synaptic levels of SV2, and resulted in reduced synaptic release probability. The overexpression phenotype parallels that seen in neurons from SV2 knockout mice, which experience severe seizures. Overexpression of SV2A also increased synaptic levels of the calcium-sensor protein synaptotagmin, an SV2-binding protein whose stability and trafficking are regulated by SV2. Treatment with levetiracetam rescued normal neurotransmission and restored normal levels of SV2 and synaptotagmin at the synapse. These results indicate that changes in SV2 expression in either direction impact neurotransmission, and suggest that levetiracetam may modulate SV2 protein interactions.

## Introduction

Levetiracetam ((S)-α-ethyl-2-oxo-pyrrolidine acetamide) represents a new class of drug for the treatment of neurological and psychiatric disorders. Currently marketed as Keppra®, levetiracetam is FDA-approved for the treatment of epilepsy (reviewed in [1]), though it also shows promise in the treatment of anxiety disorders [2], [3], [4], pain [5], [6], [7], dyskinesias [8], [9], [10], [11], [12], and post-traumatic stress disorder [3]. The protein receptor for levetiracetam is Synaptic Vesicle Protein 2A (SV2A) [13], a membrane glycoprotein specific to the secretory vesicles of neurons and endocrine cells in vertebrates [14]. SV2A is both necessary and sufficient for levetiracetam binding [13]. In addition, mice heterozygous for the SV2A gene disruption show reduced response to drug treatment [15], consistent with SV2A being required for levetiracetam action. Thus levetiracetam appears to act by modulating the action of SV2A, though its mechanism of action remains unknown.

Mammals have three SV2 genes that encode the isoforms SV2A, SV2B, and SV2C [16], [17], [18], [19]. Of these, SV2A is the most broadly expressed, and is present in essentially all neurons [20]. Notably, it is the only isoform expressed in many inhibitory, GABAergic neurons [20], [21]. SV2A is essential for survival in mice; gene disruption results in severe seizures and premature death [22], [23]. At the level of the synapse, SV2 acts as a positive modulator of calcium-dependent exocytosis. Neurons lacking SV2A or SV2A+B display reduced evoked transmitter secretion in excitatory [24], [25] and inhibitory [22], [26] neurons, as well as in cultured chromaffin cells [27]. In most systems this effect correlates with a reduction in the number of vesicles able to respond to increased presynaptic calcium (the readily releasable pool) [24], [25], [27]. Thus SV2 appears to act as a positive modulator of secretory vesicle priming in neuroendocrine cells.

Although its structural similarity to the Major Facilitator transporter family suggests that SV2 is a transporter, its demonstrated actions include regulating the expression and trafficking of the calcium binding protein synaptotagmin [28], and affecting presynaptic calcium concentrations [25]. This suggests SV2 may regulate neurotransmission indirectly by controlling the vesicle's ability to detect changes in presynaptic calcium.

Several studies report a correlation between increased SV2 expression and changes in synaptic functioning. Kindling of seizures in rats results in increased expression of several synaptic vesicle proteins including SV2 [29], [30], [31], and SV2A is at the hub of seizure-dependent changes in protein co-expression [32]. In addition, mRNA encoding SV2A is a primary target of a microRNA whose expression is sensitive to changes in synaptic activity [33]. Together these findings indicate that altered expression of SV2, and particularly SV2A, is a molecular signature of altered synaptic activity.

In animals subjected to seizure kindling protocols, treatment with levetiracetam blocks both the development of a seizure phenotype and increases in SV2 expression [31], [34]. In hippocampal slices from non-epileptic animals, treatment with levetiracetam reduces neurotransmission in response to fast stimulus trains [35], consistent with the drug blocking SV2's effects on vesicle priming. Most interestingly, the latency of drug action is much shorter when neurons are stimulated [36], suggesting that levetiracetam preferentially targets hyperactive synapses.

Here we report the effects of overexpressing SV2A in hippocampal neurons cultured to form autaptic synapses. We found that elevated expression of SV2 resulted in a neurotransmission phenotype that resembled that seen in neurons from SV2 knockout mice, suggesting that too much SV2 is as detrimental to neuronal function as too little. Because the same neurotransmission phenotype is associated with seizures in SV2A knockout mice, this offered the opportunity to assess the effects of levetiracetam under cellular conditions associated with epilepsy.

## Results

### Expression of SV2A-EGFP in wild-type neurons reduces neurotransmitter release

**Figure 1:**
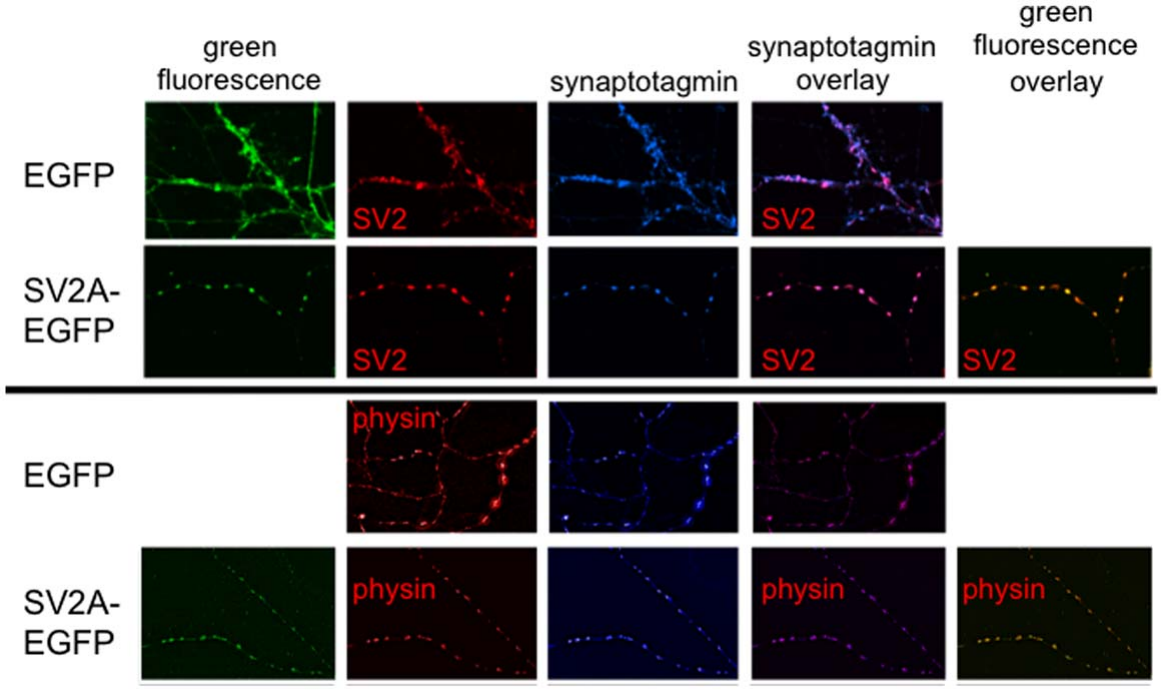
SV2A-EGFP is trafficked to presynaptic terminals. Neurons expressing either EGFP or an SV2A-EGFP fusion protein were fixed and processed for immunolabeling. In neurons expressing EGFP green fluorescence was present throughout the length of neurites, whereas in neurons expressing SV2A-EGFP green fluorescence was punctate. Immunolabeling with anti-SV2 and anti-synaptotagmin revealed that the two proteins co-localized. Immunolabeling with anti-synaptotagmin and anti-synaptophysin revealed that synaptotagmin co-localized with synaptophysin. The co-localization of these three presynaptic proteins with SV2A-EGFP indicates that the exogenous fusion protein is trafficked to presynaptic terminals.

We examined the effect of expressing **SV2A-EGFP** (SV2A with enhanced green fluorescent protein (EGFP) fused to the carboxy terminus) in wild-type autaptic cultures of hippocampal neurons as part of a larger study designed to assess the effects of mutations in SV2 [37]. SV2A-EGFP functions like native SV2, as evidenced by the fact that it restores normal neurotransmission to hippocampal neurons cultured from SV2A/B knockout mice [28], [37]. SV2A-EGFP trafficked to presynaptic terminals in wild-type neurons, where it co-localized with the synaptic vesicle protein synaptotagmin and synaptophysin (**Figure 1**).

**Figure 2:**
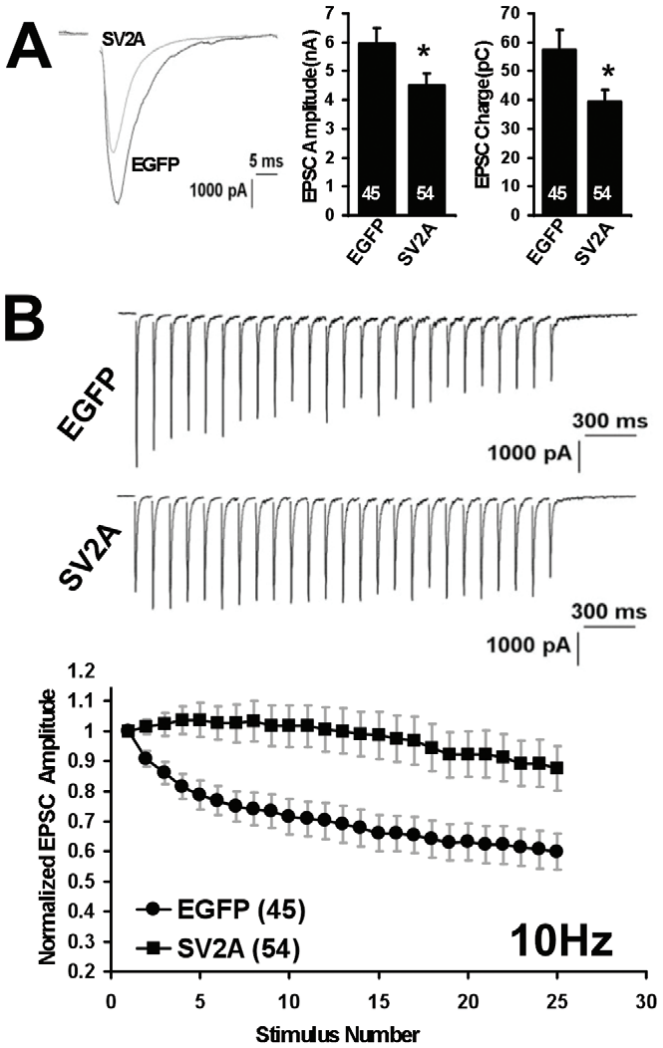
Overexpression of SV2A-EGFP in wild-type neurons results in reduced EPSC amplitude and decreased synaptic depression. ***A*** *)* Representative traces, average EPSC amplitude, and average EPSC charge transfer for cultured wild-type (**WT**) neurons expressing EGFP or SV2A-EGFP. ***B*** *)* (*top*) Representative traces of EPSCs induced by a 10 Hz, 2.5 sec stimulus in WT cells expressing EGFP or SV2A-EGFP. (*bottom*) Plot of averaged, normalized EPSC amplitudes in response to a 10 Hz stimulus train. Data are means ± SEMs. The numbers of cells recorded from is indicated in parentheses and is representative of at least 10 independent cultures. Asterisks indicate a significant difference in measurement when compared to control (student's t-test; *p<0.05).

To our surprise, expression of SV2-EGFP in wild-type hippocampal neurons resulted in a neurotransmission phenotype that looked similar to that seen in neurons cultured from SV2A/B knockout mice. As in neurons from SV2A/B knockout mice, action potential-induced excitatory postsynaptic currents (EPSC) were reduced in neurons expressing SV2A-EGFP. EPSC amplitudes were, on average, 65% the amplitude of control neurons expressing just EGFP (**Figure 2**). More importantly, the hallmark feature of the SV2 knockout phenotype, reduced synaptic depression, was also present in wild-type neurons expressing the SV2A-EGFP construct. Synaptic depression is a form of short-term plasticity that correlates with synaptic release probability [38] and is thus used to measure the ability of synapses to respond to an action potential. In neurons expressing SV2A-EGFP, responses to a 10 Hz stimulus train did not decline to the same extent as in neurons expressing EGFP. This indicates that synaptic release probability is lower when neurons express elevated levels SV2A.

### Levetiracetam restores synaptic depression in wild-type neurons expressing SV2A-EGFP

**Figure 3:**
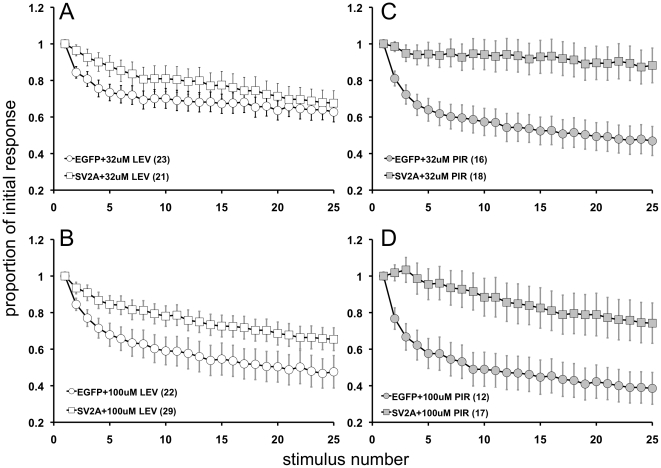
Addition of levetiracetam reverses the effect of SV2A overexpression in wild-type neurons. Responses to a 2.5 sec, 10 Hz stimulus train recorded from WT neurons expressing EGFP or SV2A-EGFP that were incubated for 6–10 hrs in (***A***) 32 µM leviteracetam, (***B***) 100 µM leviteracetam, (***C***) 32 µM piracetam, or (***D***) 100 µM piracetam. Neurons incubated in 32 µM or 100 µM leviteracetam displayed normal WT-like synaptic depression. For each neuron, responses were normalized to the first response in the stimulus train. Graphs show mean ± SEM. The numbers of cells recorded from are indicated in parentheses and are representative of 10(*A*), 6(*B*), 5(*C*), and 4(*D*) independent cultures.

Levetiracetam is reported to have multiple subtle effects on neurons, and the effective concentrations and incubation times for these different effects vary (reviewed in [39]. The ability of levetiracetam to reduce neurotransmission in quiescent neurons requires higher concentrations of drug (100 µM) and relatively long incubation times (≥3 hrs) [35], [40]. Thus to determine if levetiracetam altered the neurotransmission effects of SV2A overexpression, we examined synaptic depression, the most robust measure of the overexpression phenotype, in neurons treated with 32 uM or 100 µM levetiracetam for 1 hr or 6–10 hrs. Incubation with 100 µM levetiracetam for 6–10 hr restored normal synaptic depression in neurons expressing SV2A-EGFP (**Fig. 3B**). Treatment for 6–10 hrs with 32 µM also restored synaptic depression, though the synaptic depression was not of the same magnitude as with the higher dose (**Fig. 3A**). A 1 hr treatment did not restore normal synaptic depression (*not shown*). These results show that levetiracetam reverses the synaptic deficit produced by SV2 over-expression and that the reversal requires concentrations of levetiracetam and incubation times previously shown to be necessary for drug effects on synaptic transmission [35].

As a control, we tested the effects of piracetam, a related compound with poor anti-seizure action [41] and much lower affinity for SV2A [42]. Treatment with 32 uM piracetam for 6–10 hrs did not restore synaptic depression (**Fig. 3C**). Treatment with 100 uM piracetam for 6–10 hrs resulted in some synaptic depression (**Fig. 3D**), though less than half that seen with levetiracetam. This is consistent with the effect occurring via SV2A, which has a higher affinity for levetiracetam than piracetam.

**Figure 4:**
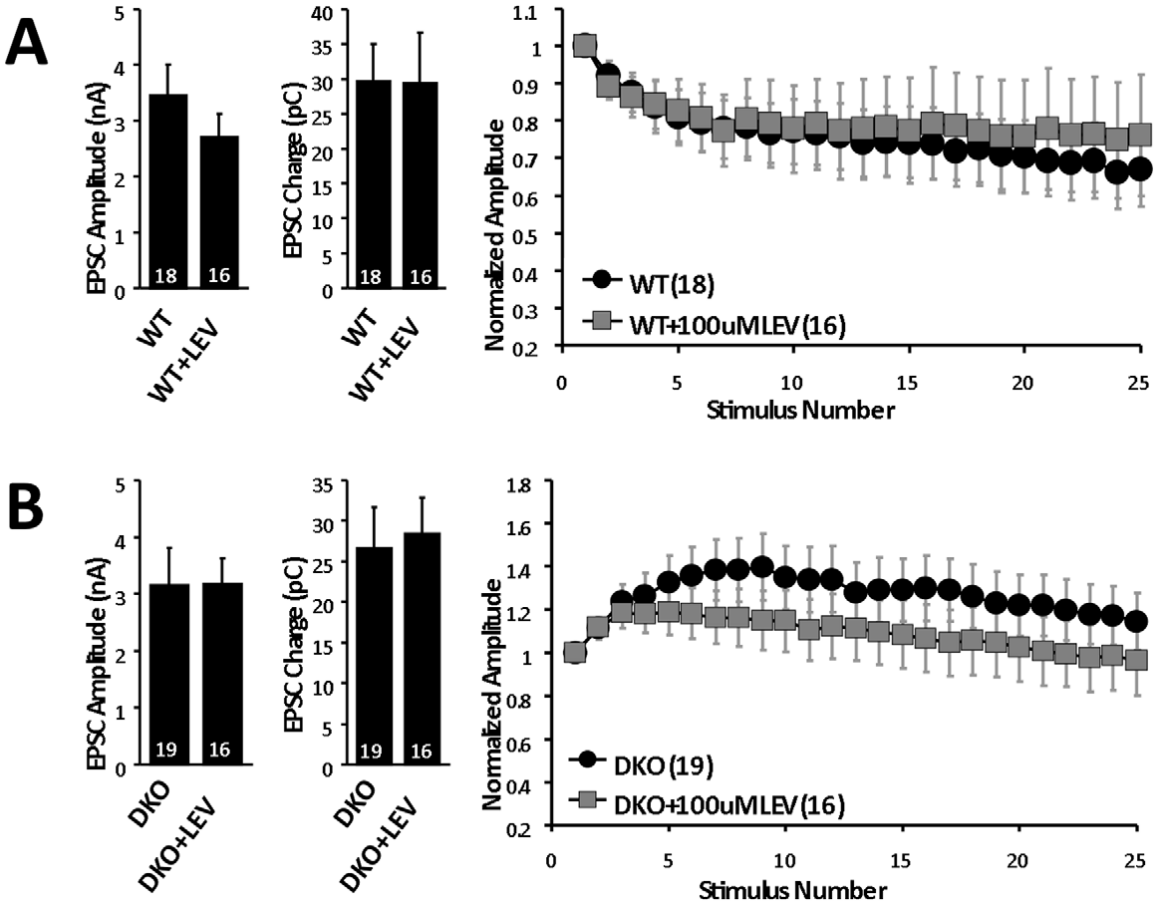
Levetiracetam has no effect on wild type neurons and does not reverse the SV2A/B knockout neurotransmission phenotype. Normalized averaged EPSC amplitude, normalized averaged EPSC charge transfer, and plot of normalized responses to a 2.5 sec, 10 Hz train recorded from neurons cultured (*A*) WT or (*B*) SV2A/B double knock-out mice (DKO). Neurons were incubated for 6–10 hrs with vehicle (water) or 100 µM levetiracetam. Shown are mean values ± SEMs. The numbers of cells recorded from are indicated in parentheses and are representative of 4(*A*), and 7(*B*) sets of cultures. Drug treatment had no significant effect on any measure.

In contrast to the studies performed in hippocampal slice preparations [35], levetiracetam did not affect neurotransmission in uninfected wild type neurons (**Fig. 4A**) nor did it restore normal EPSC amplitude or synaptic depression to neurons lacking SV2 (**Fig. 4B**). Thus overexpression of SV2A is required for levetiracetam action in this preparation.

### Levetiracetam reverses elevated SV2 at synapses expressing SV2A-EGFP

**Figure 5:**
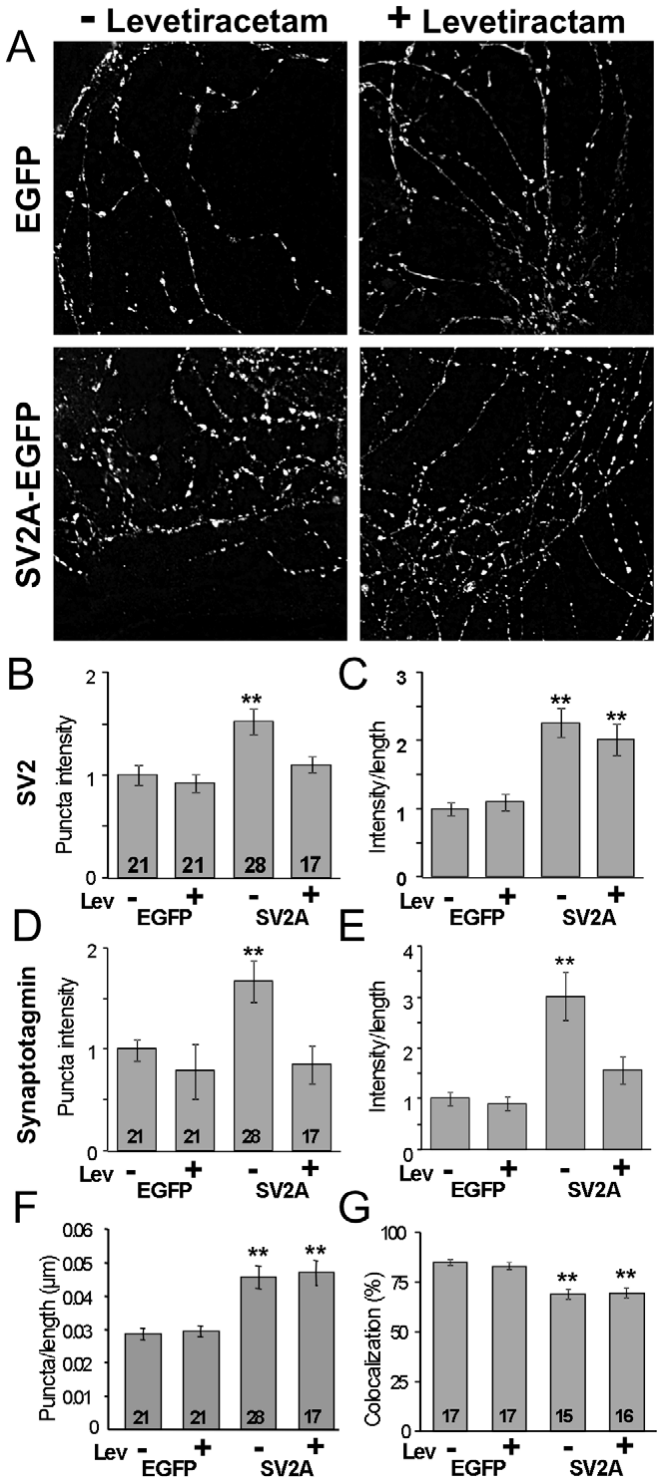
Effects of SV2-EGFP expression and levetiracetam treatment on synaptic levels of SV2 and synaptotagmin. ***A***) Neuronal processes labeled with an antibody against SV2 (all isoforms) revealed punctuate immunofluorescence along the lengths of the processes. Labeling was more intense in cells expressing SV2A-EGFP (SV2A) than in wild-type cultures infected with EGFP (EGFP-control). ***B***) Expression of SV2A-EGFP increases the amount of SV2 at synapses and treatment with levetiracetam restores normal intensity levels. Shown is a graph of the average intensity of SV2 immunolabeling in individual puncta presumed to be synapses. Incubation with 100 µM levetiracetam (Lev) for 6–8 hours restored control levels of SV2. ***C***) Expression of SV2A-EGFP increases the amount of total SV2 in neurites, and treatment with levetiracetam does not reverse the increase. Shown is a graph of the fluorescent immunolabeling of SV2 measured as the total intensity of fluorescence over the total length of neuronal processes in each image. Incubation with 100 µM levetiracetam (Lev) for 6–8 hours did not appear to affect on the amount of total SV2 expressed in the neuronal processes. ***D***) Overexpression of SV2A increases the amount of synaptotagmin at synapses and treatment with levetiracetam restores normal intensity levels. Shown is a graph of the average intensity of synaptotagmin immunolabeling in individual puncta. Incubation with 100 µM levetiracetam (Lev) for 6–8 hours restored control levels of synaptotagmin. ***E***) Overexpression of SV2A increases the total amount of synaptotagmin in neurites and treatment with levetiracetam restores normal levels. Shown is a graph of the fluorescent immunolabeling of synaptotagmin measured as in C. Incubation with 100 µM levetiracetam (Lev) for 6–8 hours restored normal synaptotagmin levels. ***F***) Graph of the number of fluorescent puncta measured per length of neurite in neurons labeled with anti-synaptotagmin. ***G***) Levetiracetam does not affect the co-localization of SV2 and synaptotagmin. Shown are graphs of the fluorescent intensity ratios of SV2 to synaptotagmin in synapses of the indicated cultures. Protein levels were measured using fluorescent immunolabeling. Bars represent the mean ± SEM. Numbers inside the bars indicate the number of images analyzed and are the same for *B* and *C*, and *D* and *E*. Data for B–E are from 4 separate cultures/replicates and for G from two cultures/replicates. To control for differences in antibody labeling between repetitions of the study, the intensity of each synaptic punctum was normalized to the average of all control (EGFP-infected) puncta within the same replicate. Asterisks indicate a significant difference in measurement when compared to EGFP-expressing neurons incubated with vehicle (one-way ANOVA followed by Fisher's LSD test; **p<0.01).

To determine the extent to which exogenous expression of SV2A-EGFP and treatment with levetiracetam affected the amount of SV2 at synapses, we measured synaptic SV2 using immunolabeling. Hippocampal neurons expressing either EGFP or SV2A-EGFP were treated with vehicle (water) or 100 µM levetiracetam for 6 hours, after which they were fixed, permeabilized and reacted with an anti-SV2 monoclonal antibody [14] that recognizes all three isoforms of SV2. Images were collected on a Deltavision deconvoluting microscope using identical exposure times and analyzed as described under *Methods*. Both the total SV2 immunoreactivity per length of neurite and average immunoreactivity intensity at synaptic puncta were quantified. We found that expression of SV2A-EGFP produced a 1.5-fold increase in the average intensity (total puncta intensity/puncta area) of SV2 fluorescent puncta, which indicates that there was more SV2 per synapse in neurons expressing exogenous SV2 than neurons infected with EGFP lentivirus alone. Incubation with levetiracetam reduced this to control levels (**Figs. 5A, B**). Interestingly, while expression of SV2A-EGFP led to a ∼2-fold increase in the total amount of SV2 per unit length of neurite, this increase was not affected by treatment with levetiracetam (**Fig. 5C**). Together these findings suggest that levetiracetam influences the localization of SV2 at the synapse rather than its overall expression or turnover.

Loss of SV2 results in a significant decrease in the expression levels of synaptotagmin [28], [43], [44], suggesting that synaptotagmin expression/stability depends on SV2. To determine whether overexpression of SV2 impacts synaptotagmin expression, we assessed the effect of SV2A-EGFP expression on synaptotagmin levels. In cells expressing SV2A-EGFP we observed a ∼2-fold increase in the average intensity of anti-synaptotagmin labeled puncta and a ∼3-fold increase in the total amount of synaptotagmin (immunoreactivity per unit length of neurite) (**Figure 5D–E**). Both measures returned to levels seen in control neurons when cultures were incubated with 100 µM levetiracetam for 6 hours. Thus levetiracetam restored both total and synaptic levels of synaptotagmin.

Expression of SV2A-EGFP also increased the density of puncta labeled with anti-synaptotagmin, suggesting that elevated SV2 levels may lead to more synapses. This increase was not affected by treatment with levetiracetam, however (**Fig. 5F**), so it is not clear that an increase in the number of puncta represents an increase in functional synapses.

We also assessed the overlap of SV2 and synaptotagmin at the synapse to determine if either overexpression and/or treatment with levetiracetam affected protein co-localization at the synapse. Cultures were co-labeled with anti-SV2 and anti-synaptotagmin and the fluorescent intensity of SV2 to synaptotagmin was expressed as a ratio for each punctum. Expression of SV2A-EGFP resulted in a small but significant decrease in the co-localization of the two proteins, and this decrease was not affected by treatment with levetiracetam (**Figure 5G**).

In summary, we find that levetiracetam treatment of neurons overexpressing SV2A 1) decreased the amount of both SV2 and synaptotagmin at synapses (Figures 4B and 4D), 2) decreased total synaptotagmin/length of neurite but did not restore lower levels of total SV2 (Figures 4C and 4E), and 3) did not alter the ratio of SV2/tagmin at synapses. Considered together these findings are consistent with the interpretation that the amount of synaptotagmin at synapses tracks the amount of SV2, and levetiracetam results in a reduction in the amount of both proteins available to support calcium-triggered exocytosis. The fact that both increased and decreased levels of SV2 and synaptotagmin are associated with reduced release probability suggests that optimal levels of these proteins are required for normal neurotransmission, a conclusion consistent with the recent observation that the number of SV2s and synaptotagmins per vesicle is tightly regulated [45].

## Discussion

The results presented here demonstrate that overexpression of SV2A produces abnormal neurotransmission that can be rescued by treatment with levetiracetam. Because expression of SV2A-EGFP rescues normal neurotransmission in neurons cultured from SV2A/B knockout mice [28], [37], the effects of overexpression are not likely to be a dominant negative effect of expressing a fusion protein. Rather they appear to be due to effects of excess SV2. We note that like SV2, overexpression of the SNARE-binding protein, complexin, also produces a decrease in synaptic release probability that resembles that seen in complexin knockout mice [46], [47], [48], [49], [50]. Thus, synaptic functioning can be regulated by both increases and decreases in protein expression, which suggests that proteins at the synapse form complexes that rely on precise stoichiometry in expression levels.

The reduced synaptic release probability we measured in neurons overexpressing SV2 is nearly identical to that seen in neurons from SV2A/B knockout mice, which experience severe seizures [22], [23]. Viewed in combination with the results presented here, it appears that changes in SV2 expression in either direction have a similar impact on synaptic functioning. Indeed, both increases and decreases in SV2 expression have been reported to be associated with the presence of seizues. SV2 expression increases with kindling of seizures, and both the appearance of seizures and elevated SV2 expression are reversed by treatment with levetiracetam [29], [31]. In contrast, qualitative analyses of expression reveal decreased SV2A expression in temporal lobe epilepsy [51]. Therefore both increased and decreased SV2 may contribute to epilepsy.

A 6–10 hr treatment with levetiracetam reversed the effects of SV2 overexpression. The requirement for longer treatment times was first reported by Yang et al., who showed that levetiracetam-mediated reduction in synaptic transmission required prolonged presence of the drug [35], [40]. Together with the observation that SV2 contributes to the number of assembled SNARE complexes [27], the delayed action of levetiracetam suggests that it (and therefore SV2) influences vesicle priming prior to the penultimate stages of vesicle fusion. Therefore, drug effects will occur after previously primed vesicles are expended. Consistent with this interpretation, synaptic activity decreases the latency of levetiracetam effects in hippocampal slices [36].

Levetiracetam had no effect on neurons that lacked SV2, consistent with the conclusion that the drug acts by binding SV2. Because SV2 expression is limited to presynaptic terminals, this means that levetiracetam affects presynaptic events that regulate synaptic vesicle release.

Given that levetiracetam restored the concentration of SV2 at the synapse (average labeling intensity per synapse area) to control levels without affecting total SV2 per length of neurite, it is most likely that levetiracetam affects the ability of SV2 to concentrate in synapses. This is most consistent with the drug altering SV2's ability to bind to proteins that influence protein trafficking or localization, for example binding to clathrin adaptor proteins or proteins of the cytoskeleton.

The effects of levetiracetam reported here, in combination with the observation that levetiracetam blocks kindling-induced increases in SV2 [34], suggest that levetiracetam may act by reversing the effects of increased SV2 expression. Because SV2 is part of a protein complex [52], one possible mechanism of drug action is that it inhibits SV2 protein interactions. In the case of SV2 overexpression, levetiracetam may inhibit inappropriate interactions that occur when SV2 is over-abundant. What is clear is that levetiracetam's action on protein levels at synapses represents a novel drug action. Future work into the mechanisms by which levetiracetam produces the effects reported here will provide insight into the etiology of nervous system disorders that are based in aberrant protein expression/function.

## Materials and Methods

### SV2 knock-out mice

The generation of SV2A and SV2B knockout mice was reported previously [22], [44]. These animals were used to obtain two lines of breeders that were SV2A+/−B+/+ or SV2A+/−B−/−. Littermate SV2A+/+B+/+ and SV2A−/−B+/+ mice were obtained by breeding SV2A+/−B+/+ animals. Similarly, SV2A+/+B−/− and SV2A−/−B−/− littermates were obtained by breeding SV2A+/−B−/− animals. All animals were 99.99% C57BL/6. Cultures were generated from mice at postnatal day 0–2. SV2A genotype was determined by PCR before culturing neurons.

The University of Washington IACUC, protocol number 2881-01, approved the use of mice for these studies.

### Constructs

Lentiviral constructs were designed to encode rat SV2A protein with enhanced-GFP (EGFP) on the carboxy terminus. The resulting fusion protein serves as a real time visual reporter of infection and proper protein trafficking. A control vector was generated encoding EGFP alone. The SV2A-EGFP or EGFP cDNA was then subcloned into the Lentiviral transfer vector pRRL-cPPT-CMV-X-PRE-SIN [53]. Lentiviral helper plasmids (pLP1, pLP2, pLP/VSVG) were from the Virapower packaging mix (Invitrogen).

### Cell culture

Primary cultures of hippocampal neurons were prepared as previously described [24]. Hippocampi were isolated and the dentate gyrus was removed. The hippocampi were then incubated in papain, mechanically dissociated, and plated at a density of 2000–3000 cells/cm2 on coverslips containing microislands of astrocytes. Astrocyte cultures were derived from wild-type mice. Levetiracetam or piracetam (Sigma Chemical Company) were dissolved in sterile water and added to cultures as a 500× stock solution. Cultures were infected with Lenti virions 1–3 days after plating.

### Electrophysiology

Whole-cell voltage-clamp recordings were obtained from neurons on single-neuron islands as previously reported [24] except that recordings were performed at 21–23°C. Recordings were performed 10–17 d after plating. For EPSC amplitude measurements, three responses were obtained and averaged for each cell.

### Data analysis and statistics

Action potential-evoked EPSCs were analyzed with custom software written in Visual C# (Microsoft, Redmond, WA) as previously described [24]. EPSC amplitudes were determined by subtracting a baseline current (obtained by averaging the period 3 ms before the stimulus) from the peak current of the EPSC. Responses less than 1 nA were not included. Statistical analyses were performed with Microsoft Excel. Reported data are mean ± SEM. In all cases, t-tests were two-tailed unpaired tests assuming unequal variances. Immunocytochemistry studies were analyzed using one-way ANOVA followed by Fisher's LSD test.

### Indirect Immunocytochemistry

For determining the subcellular localization of SV2, cultured neurons were fixed with 4% paraformaldehyde (EM sciences) in phosphate buffered saline (PBS) for 20 min at room temperature. Cells were then washed with PBS containing glycine (0.1 M). PBS supplemented with 2% normal goat serum, 0.1% bovine serum albumin, and 0.4% saponin was used for 10 min to block nonspecific binding and permeabilize cells. The cells were then incubated with primary antibody against SV2 [14] or synaptotagmin [52] overnight at 4°C. Following washout of primary antibodies, cells were incubated with Alexa-Fluor 568 or Alexa-Fluor 657-conjugated secondary antibodies (Invitrogen, 1∶2000) for 1 h. Parallel control experiments were performed in which primary antibodies were omitted to test for the nonspecific binding of secondary antibodies. Neurons were imaged at the W. M. Keck Center for Advanced Studies in Neural Signaling using a DeltaVision microscope (Applied Precision, Issaquah, WA) with a 60× oil immersion objective, Sedat quad filter set (Chroma Technologies), Photometrics CH350 CCD camera using Softworx 2.50. A z-series of images was collected at a resolution of 0.113 µm/pixel and deconvolved. A representative z-plane was chosen for analysis. Images were processed using MetaMorph (Molecular Devices, Sunnyvale, CA) to measure dendritic length and to detect and count fluorescent puncta larger than four pixels. Colocalization of SV2A and synaptotagmin labeling was measured using MetaMorph. To assess co-localization The percentage of synaptotagmin-positive cell area that was also positive for synaptophysin was assessed. All imaging data were background subtracted using fluorescence emission from a region of the coverslip containing no cells.
